# Detection and characterization of bacterial nucleic acids in culture-negative synovial tissue and fluid samples from rheumatoid arthritis or osteoarthritis patients

**DOI:** 10.1038/s41598-018-32675-w

**Published:** 2018-09-24

**Authors:** Yan Zhao, Bin Chen, Shufeng Li, Lanxiu Yang, Dequan Zhu, Ye Wang, Haiying Wang, Tao Wang, Bin Shi, Zhongtao Gai, Jun Yang, Xueyuan Heng, Junjie Yang, Lei Zhang

**Affiliations:** 1 College of Life Science, Shandong Normal University, Jinan, 250014 China; 2 Shandong Medicinal and Biotechnology Centre, Shandong Academy of Medical Sciences, Jinan, 250062 China; 3 Department of Orthopedics, Qianfoshan Hospital Affiliated to Shandong University, Jinan, 250014 China; 4 Guoyitang Hospital, Jinan, 250000 China; 5 Microbiological Laboratory, Linyi People’s Hospital, Linyi, 276003 China; 6 Qingdao Human Microbiome Center, The Affiliated Central Hospital of Qingdao University, Qingdao, 266000 China; 7 Shandong Children’s Microbiome Center, Qilu Children’s Hospital of Shandong University, Jinan, 250001 China; 8 College of Life Science, Qilu Normal University, Jinan, 250200 China; 9 Shandong Institutes for Food and Drug Control, Jinan, 250101 China; 10 Beijing Advanced Innovation Center for Big Data-Based Precision Medicine, School of Chemistry and Environment, Beihang University, Beijing, 100191 China

## Abstract

Human intestinal microbes can mediate development of arthritis – Studies indicate that certain bacterial nucleic acids may exist in synovial fluid (SF) and could be involved in arthritis, although the underlying mechanism remains unclear. To characterize potential SF bacterial nucleic acids, we used 16S rRNA gene amplicon sequencing to assess bacterial nucleic acid communities in 15 synovial tissue (ST) and 110 SF samples from 125 patients with rheumatoid arthritis (RA) and 16 ST and 42 SF samples from 58 patients with osteoarthritis (OA). Our results showed an abundant diversity of bacterial nucleic acids in these clinical samples, including presence of *Porphyromonas* and *Bacteroides* in all 183 samples. *Agrobacterium*, *Comamonas*, *Kocuria*, *Meiothermus*, and *Rhodoplanes* were more abundant in synovial tissues of rheumatoid arthritis (STRA). *Atopobium*, *Phascolarctobacterium*, *Rhodotorula mucilaginosa*, *Bacteroides uniformis*, *Rothia*, *Megasphaera*, *Turicibacter*, *Leptotrichia*, *Haemophilus parainfluenzae*, *Bacteroides fragilis*, *Porphyromonas*, and *Streptococcus* were more abundant in synovial tissues of osteoarthritis (STOA). *Veillonella dispar*, *Haemophilus parainfluenzae*, *Prevotella copri* and *Treponema amylovorum* were more abundant in synovial fluid of rheumatoid arthritis (SFRA), while *Bacteroides caccae* was more abundant in the synovial fluid of osteoarthritis (SFOA). Overall, this study confirms existence of bacterial nucleic acids in SF and ST samples of RA and OA lesions and reveals potential correlations with degree of disease.

## Introduction

RA is a synovitis-based systemic autoimmune disease whose etiology remains elusive although both genetic and environmental factors play an important role in disease pathogenesis^1,2^. The pathogenesis of OA is also unclear, although various parameters, including age, infection, inflammation, injury, extra-articular deformity, joint instability, environmental factors, estrogen, excess weight, excessive exercises, genetics, and diet may be risk factors^3,4^. Although infection has not been considered a major factor in the pathogenesis of RA and OA, discovery of inflammatory cytokines and the currently unsatisfactory therapeutic options have led us to rethink the role of bacterial infection in the development and pathogenesis of RA and OA.

Recent data indicate a role for human microbiota in the pathology of inflammatory arthritis, as mucosa exposed to high loads of bacterial antigens (such as in intestines) may break through the first immune resistance of rheumatoid arthritis, psoriatic arthritis, and related diseases. For example, Wu *et al*. found that sterile conditions significantly abate spontaneous arthritis in K/BxN mice^5^. Further, injection of filamentous bacteria induces murine autoimmune responses and affects the role of T17 cells, with bacteria influencing IL17 secretion and increasing production of antibodies, thus resulting in arthritis. In a specific pathogen-free environment, administration of *E*. *coli* DH5a enemas to Kunming mice directly induces arthritic symptoms, with joints showing invasion of inflammatory cells^6^.

In humans, Scher *et al*. used 16S rRNA gene sequencing of stool samples from 44 RA patients to show that *Prevotellacopri*, a human intestinal bacterium, may mediate development of RA^7,8^. Further, Zhang *et al*. used shotgun metagenomic sequencing of saliva, dental plaque and fecal samples to show that RA patients lack *Haemophilus* species, and that their abundance is inversely proportional to RA autoimmune antibody titers^9^. However, *Lactobacillus salivarius* is significantly enriched in dental plaque, saliva, and fecal samples of RA patients, especially those with highly active disease.

Work by Cukrowska *et al*. indicates that intestinal microbiota do not induce immune responses under a balanced state^10^. Instead, as a result of dysregulation of intestinal microbiota, normal microbiota can act as an external antigen to stimulate lymphocyte proliferation and differentiation. Activated lymphocytes can then release various cytokines, such as IL-1, IL-6, IL-17, and TNF-α. IL-1 and TNF-α prompt white blood cells to accumulate in the articular cavity and stimulate production of small molecule inflammatory mediators, thereby leading to cartilage damage and changes in bone.

Early studies found that *Clostridium perfringens* is significantly more abundant in the feces of RA patients than control samples and is associated with the degree of RA activity^11^. Further, a DNA-RNA hybrid comparative study of fecal samples from early RA patients (≤6 months) and fibromyalgia patients found that RA patients have significantly decreased abundance of fecal *Bifidobacteria*, *Bacteroides*, fragile *Bacillus subtilis*, *Mycobacterium faecalis*, *Clostridium difficile*, and other species, compared to controls^12^.

Most RA studies have analyzed fecal samples to show that dysregulation of intestinal microbiota is involved in RA pathogenesis, although few studies have examined the intestinal microbiota of OA patients. Bacterial peptidoglycans can be detected in joint samples from OA patients^13^, and peptidoglycan, combined with autoantibodies, can activate extra-articular reactive B cells^14^. In addition, mycoplasma may also contribute to the development of chronic inflammation in arthritis. PCR analysis of 16S rRNA from arthritis patients identified *Mycoplasma pneumoniae* in the synovial fluid of 19/24 RA patients (79%) and 8/10 OA patients (80%), but not in control samples from trauma patients^15^.

To date, no literature has analyzed potential intra-articular bacterial nucleic acids from *in situ* lesions using high-throughput techniques or has evaluated involvement of bacterial nucleic acids in RA and OA lesions from the perspective of bacterial infection. Therefore, we collected 31 ST (15 STRA and 16 STOA) samples and 152 SF (110 SFRA and 42 SFOA) samples and used 16S rRNA gene sequencing to evaluate the *in situ* existence of microbiota in lesions and their potential involvement in the development and progression of arthritis.

## Results

### Detection of bacterial nucleic acids and characterization of microbiota in RA and OA synovial samples

**Figure 1:**
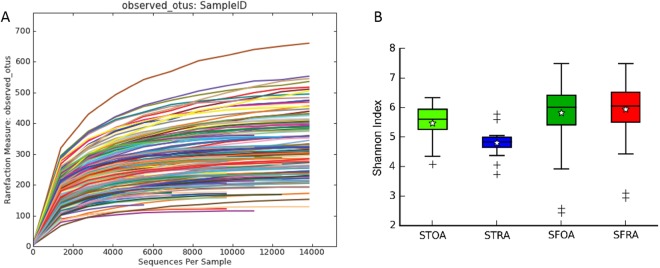
Rarefaction analysis of the microbe species from synovial tissue and synovial fluid of Rheumatoid arthritis or Osteoarthritis. (**A**) Rarefaction curve; (**B**) Shannon index. STOA = synovial tissue from osteoarthritis, STRA = synovial tissue from rheumatoid arthritis, SFOA = synovial fluid from osteoarthritis, SFRA = synovial fluid from rheumatoid arthritis.

Figure 1A displays all samples with V1-V2 sequencing using 16S rRNA, with operational taxonomic units (OTUs) based on 97% sequence similarity. We obtained 290 ± 116 OTUs per sample. Alpha rarefaction plots of observed species were constructed to determine that adequate sequence coverage was obtained to reliably describe the full diversity present in these samples.

The Shannon index was used to assess and compare α-diversity of samples of SFOA, STOA, SFRA, and STRA. The data in Fig. 1B illustrates that the α-diversity of STOA was significantly higher than that of STRA samples (*P* = 0.00399 STOA vs STRA), and the α-diversity of SFRA was significantly higher than that of SFOA samples (*P* = 0.03704 SFRA vs SFOA). In addition, the α-diversity of SFRA was significantly higher than that of STRA samples (*P* = 2.085e-06 SFRA vs STRA). Although α-diversity of SFOA was higher than that of STOA samples, the difference was not significant (*P* = 0.1689 SFOA vs STOA).

**Figure 2:**
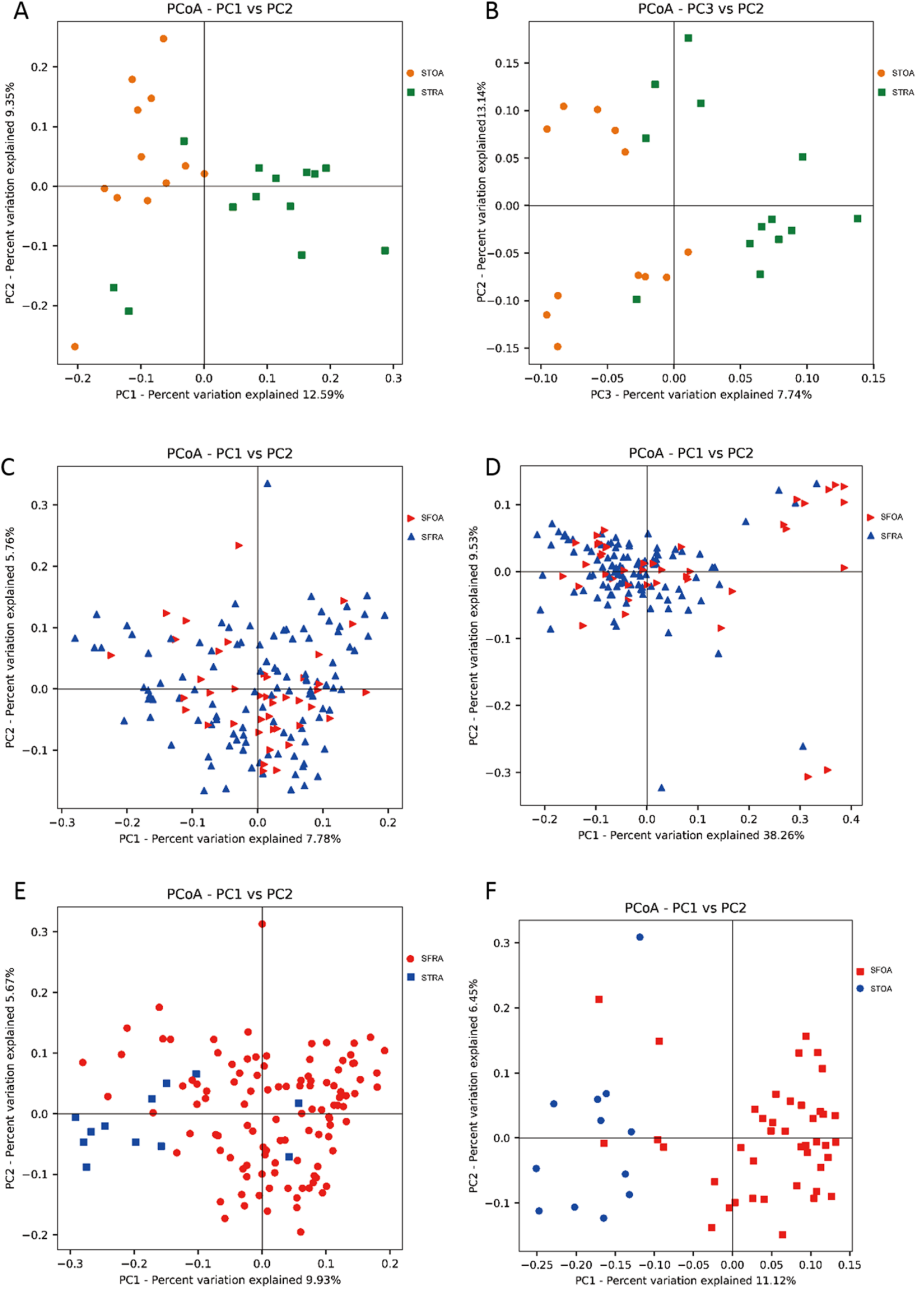
Principal coordinate analysis (PCoA) of unweighted and weighted UniFrac distances of all samples. (**A**) PCoA unweighted UniFrac PC1 vs PC2, STOA = synovial tissue from osteoarthritis, STRA = synovial tissue from rheumatoid arthritis. (**B**) PCoA weighted UniFrac PC3 vs PC2, STOA = synovial tissue from osteoarthritis, STRA = synovial tissue from rheumatoid arthritis. (**C**) PCoA unweighted UniFrac PC1 vs PC2, SFOA = synovial fluid from osteoarthritis, SFRA = synovial fluid from rheumatoid arthritis. (**D**) PCoA weighted UniFrac PC1 vs PC2, SFOA = synovial fluid from osteoarthritis, SFRA = synovial fluid from rheumatoid arthritis. (**E**) PCoA unweighted UniFrac PC1 vs PC2, SFRA = synovial fluid from rheumatoid arthritis, STRA = synovial tissue from rheumatoid arthritis. (**F**) PCoA weighted UniFrac PC1 vs PC2, SFOA = synovial fluid from osteoarthritis, STOA = synovial tissue from osteoarthritis.

For compositional changes in microbiota, we performed principal coordinate analysis (PCoA) of unweighted and weighted UniFrac distances on all samples. Figure 2A displays unweighted UniFrac of synovial tissue samples, which indicates significant aggregations in STRA and STOA samples (*P* = 0.0001, r = 0.287, ANOSIM). Figure 2B displays weighted UniFrac of synovial tissue samples, which indicate significant aggregations in STRA and STOA samples (*P* = 0.04, r = 0.1226, ANOSIM). However, as shown in Fig. 2C and D, unweighted (*P* = 0.30, r = 0.0205, ANOSIM) UniFrac of synovial fluid samples showed no significant aggregations in SFRA and SFOA samples, but weighted (*P* = 0.0013, r = 0.1605, ANOSIM) UniFrac PCoA showed significant aggregations in SFRA and SFOA. At the same time, as shown in Fig. 2E and F, unweighted (*P* = 0.0001, r = 0.5882, ANOSIM) UniFrac of RA samples showed significant aggregations in STRA and SFRA samples, while analysis of OA samples showed significant aggregations in STOA and SFOA samples as well (*P* = 0.0001, r = 0.661, ANOSIM).

### Comparison of bacterial community composition in RA and OA synovial samples

**Figure 3:**
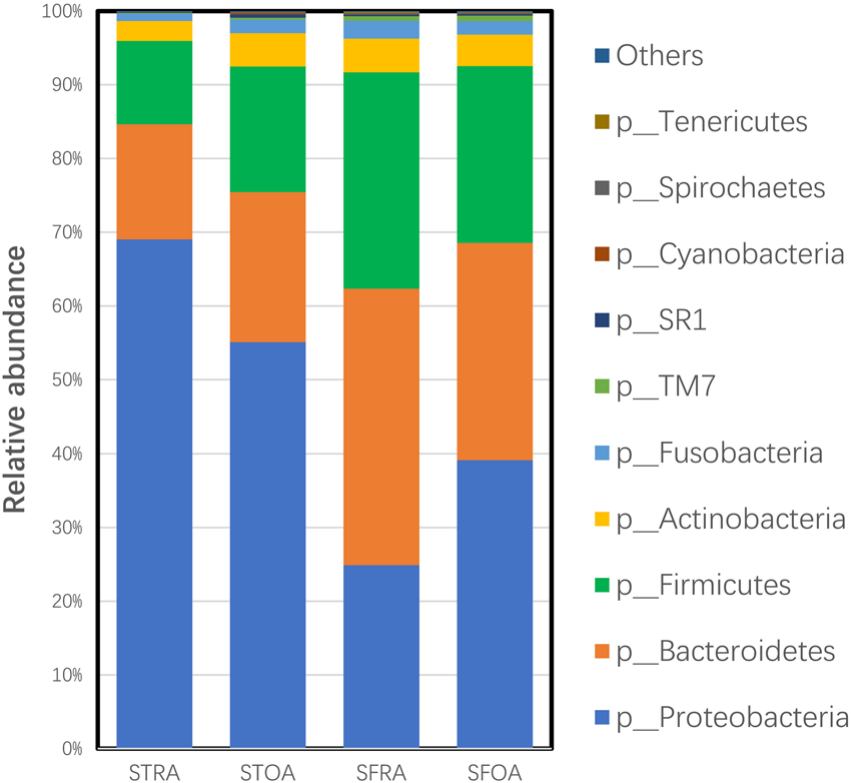
Microflora distributions of the microbe species from synovial tissue and synovial fluid of Rheumatoid arthritis or Osteoarthritis in the phylum level. STRA = synovial tissue from rheumatoid arthritis. STOA = synovial tissue from osteoarthritis, SFRA= synovial fluid from rheumatoid arthritis. SFOA = synovial fluid from osteoarthritis.

Phylum-based microbial components of RA and OA were similar, with differences in the ratio of certain types of bacteria. As shown in Fig. 3, the most abundant phyla in all synovial samples were *Proteobacteria* (STRA, 69.0%; STOA, 55.1%; SFRA, 24.9%; SFOA, 39.1%), *Bacteroidetes* (STRA, 15.6%; STOA, 20.4%; SFRA, 37.4%; SFOA, 29.4%), and *Firmicutes* (STRA, 11.3%; STOA, 17.0%; SFRA, 29.4%; SFOA, 24.0%).

**Figure 4:**
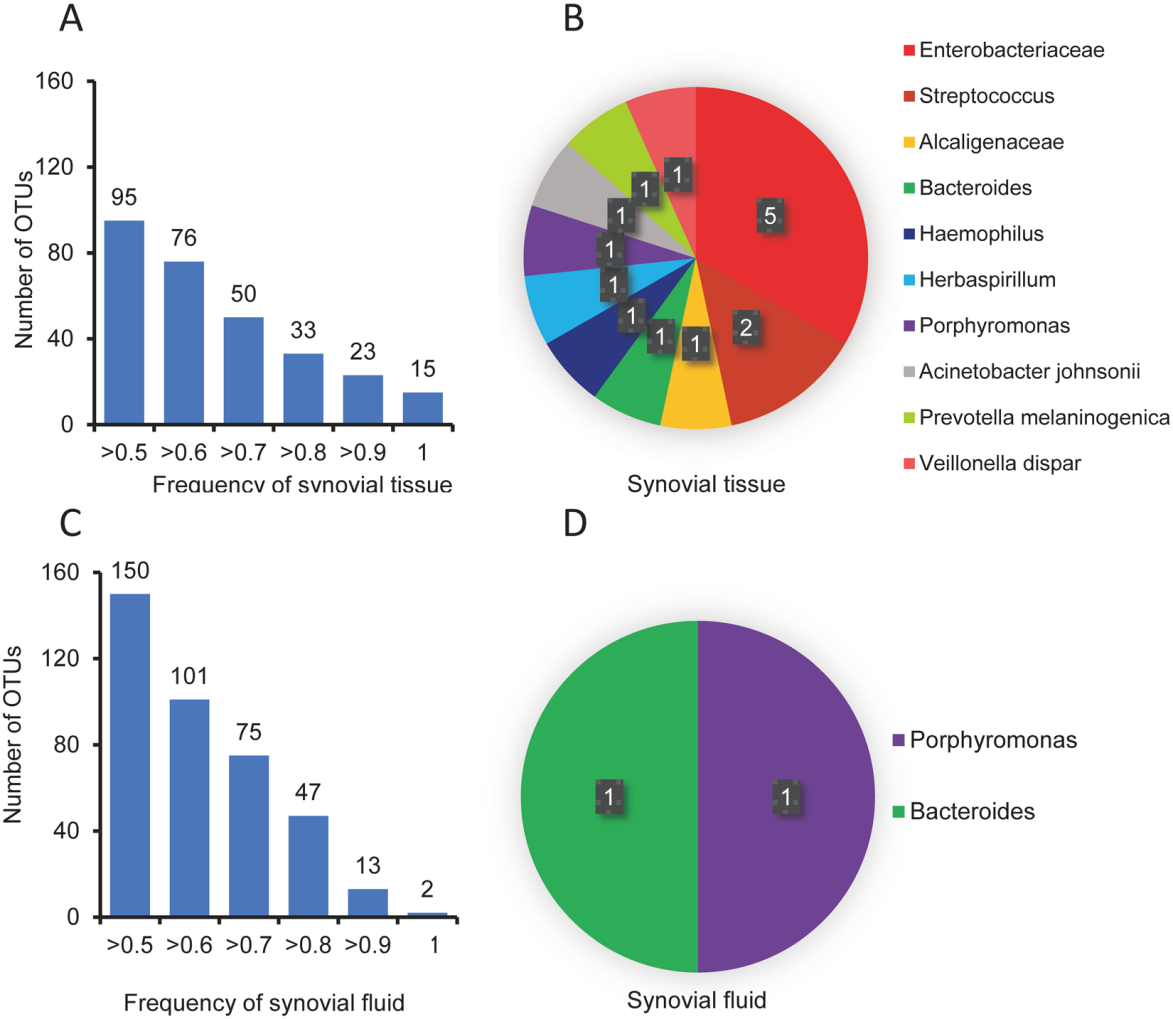
OTU analysis of all synovial samples. (**A**) OTU frequency histogram for all synovial tissue samples. (**B**) Pie chart of shared OTUs in all synovial tissue samples. (**C**) OTU frequency histogram for all synovial fluid samples. (**D**) Pie chart of shared OTUs in all synovial fluid samples.

We compared OTUs in ST samples and found that the frequencies of 95 OTUs were >0.5, as shown in Fig. 4A. There are species in all ST samples (15 OTUs), including *Enterobacteriaceae*, *Streptococcus*, *Alcaligenaceae*, *Bacteroides*, *Haemophilus*, *Herbaspirillum*, *Porphyromonas*, *Acinetobacter johnsonii*, *Prevotella melaninogenica*, and *Veillonella dispar*. The results are displayed in Fig. 4B. As shown in Fig. 4C, the frequencies of 150 OTUs in SF were >0.5. There are species in all SF samples (2 OTUs), including *Porphyromonas* and *Bacteroides*. The results are displayed in Fig. 4D. *Porphyromonas* and *Bacteroides* were present in all ST samples and SF samples.

**Figure 5:**
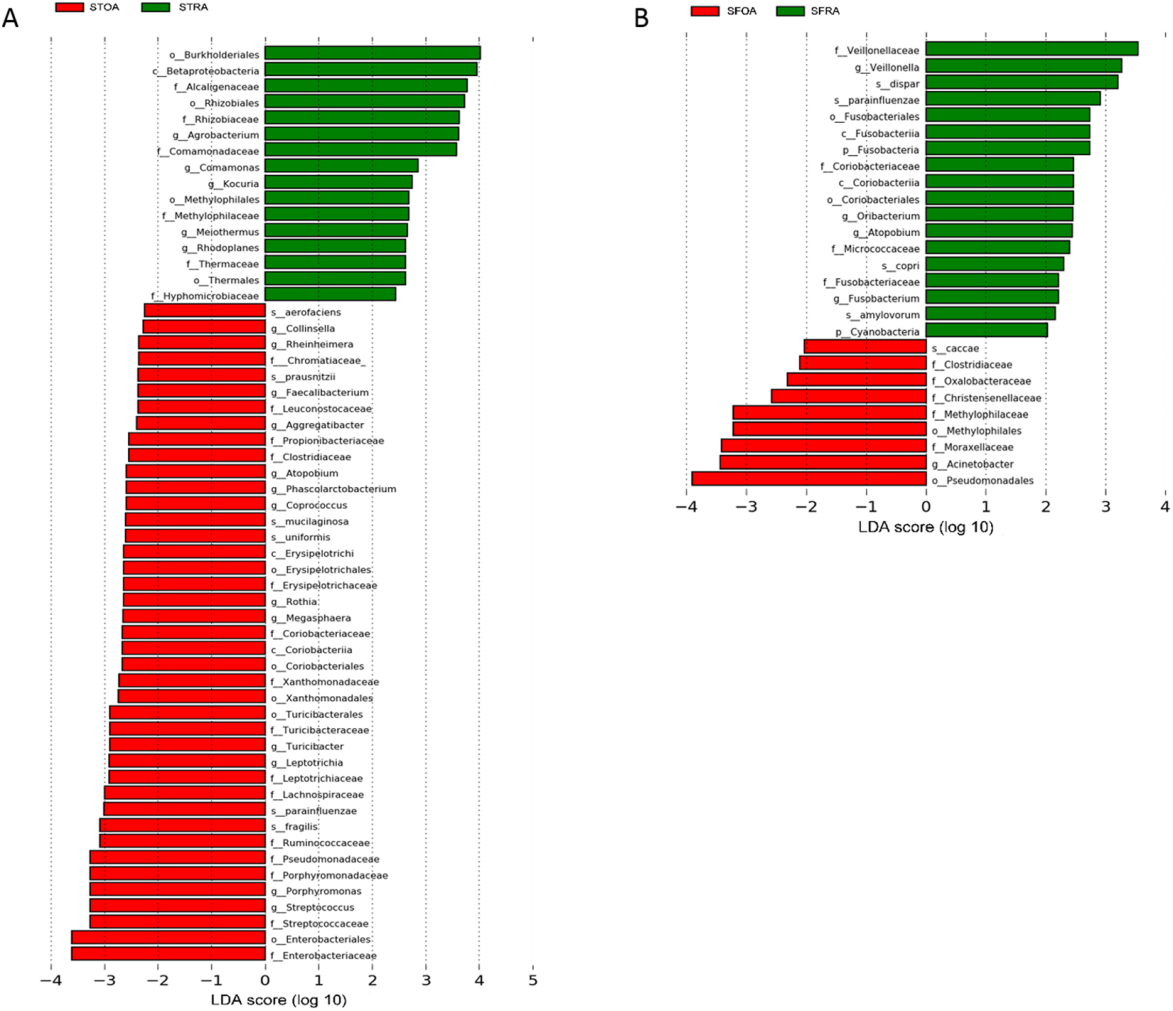
Histogram of linear discriminant analysis (LDA) scores for microbes differentially abundant in Rheumatoid arthritis or Osteoarthritis. (**A**) Histogram plot of LDA scores for microbes in STOA compared to STRA groups. (**B**) Histogram plot of LDA scores for microbes in SFOA compared to SFRA groups. RA-enriched microbes are indicated with a positive LDA score, while OA-enriched microbes are indicated with a negative score. LDA score indicates the effect size and ranking of each differentially abundant taxon. STRA = synovial tissue from rheumatoid arthritis. STOA = synovial tissue from osteoarthritis, SFRA = synovial fluid from rheumatoid arthritis. SFOA = synovial fluid from osteoarthritis.

LEfSe (Linear discriminant analysis effect size) was performed to further explore microbial differences in ST and SF of RA and OA patients. In LEfSe, linear discriminant analysis (LDA) was adopted to evaluate the data and effects on significantly different species. LEfSe identified 48 characteristic features (LDA score > 2; α < 0.05) that significantly differed in relative abundance between STOA and STRA. *Agrobacterium*, *Comamonas*, *Kocuria*, *Meiothermus*, and *Rhodoplanes* were concentrated in STRA; and *Atopobium*, *Phascolarctobacterium*, *Rhodotorula mucilaginosa*, *Bacteroides uniformis*, *Rothia*, *Megasphaera*, *Turicibacter*, *Leptotrichia*, *Haemophilus parainfluenzae*, *Bacteroides fragilis*, *Porphyromonas*, and *Streptococcus* were concentrated in STOA. The results are displayed in Fig. 5A. Further, we identified 27 characteristic features (LDA score > 2; α < 0.05) that significantly differed in relative abundance between SFOA and SFRA. *Veillonella dispar*, *Haemophilus parainfluenzae*, *Prevotella copri*, and *Treponema amylovorum* were concentrated in SFRA, while *Bacteroides caccae* was more abundant in SFOA. The results are displayed in Fig. 5B.

### PICRUSt algorithm prediction of potential microbiota functions

**Figure 6:**
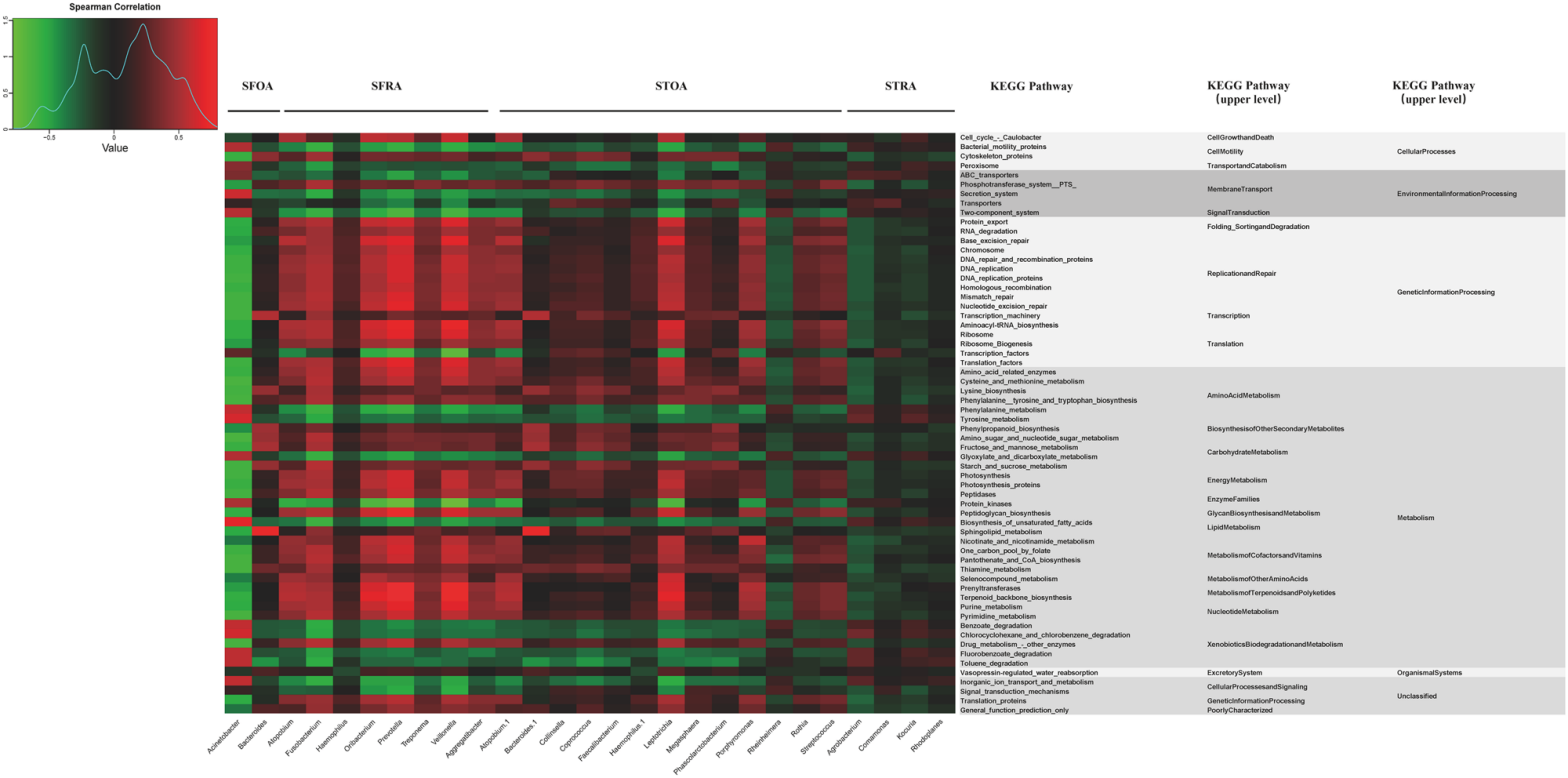
Hierarchically clustered heatmap analysis of KEGG pathways in synovial tissue and synovial fluid of Rheumatoid arthritis or Osteoarthritis. The relative values for bacteria are indicated by color intensity with the legend indicated at the top-left corner of the figure. KEGG = Kyoto Encyclopedia of Genes and Genomes. STRA = synovial tissue from rheumatoid arthritis. STOA = synovial tissue from osteoarthritis, SFRA = synovial fluid from rheumatoid arthritis. SFOA = synovial fluid from osteoarthritis.

PICRUSt (Phylogenetic Investigation of Communities by Reconstruction of Unobserved States) algorithm was adopted to predict microbiota functions. Among the 277 tested Kyoto Encyclopedia of Genes and Genomes (KEGG) pathways, 11 pathways differed between SFRA and SFOA (LDA > 2), and 55 pathways differed between STRA and the STOA (LDA > 2), including pathways related to metabolism, genetic information processing, environmental information processing, cellular processes, and body systems. The results are displayed in Fig. 6.

We also evaluated the relationship between genus and relevant metabolic pathways for SFRA and SFOA and for STRA and STOA groups. Microbial genera of the SFOA group and pathways related to processing of genetic information (folding, sorting and degradation, repair and replication, translation, and transcription) were negatively correlated with metabolism-related pathways (metabolism of amino acids, carbohydrates, energy, coenzyme factors and vitamins, terpenoids and ketones, and nucleotides), while genera of the SFRA group had a positive correlation. However, genera of the SFOA group were positively correlated, while those of the SFRA group were negatively correlated, with transcription factors. Microbial genera of the STOA group and pathways related to processing of genetic information (folding, sorting and degradation, repair and replication, translation, and transcription) were positively correlated with metabolic-related pathways (metabolism of amino acids, carbohydrates, energy, coenzyme factor and vitamin, terpenoids and ketones, and nucleotides), cytoskeletal proteins, and drug metabolism-related enzymes, while genera of the STRA group had a negative correlation. However, genera of the STOA group were negatively correlated, while genera of the STRA group was positively correlated, with degradation and metabolism-related pathways of foreign matters, phenylalanine metabolism, dicarboxylic acid metabolism, unsaturated fatty acid biosynthesis, and protein kinase.

### Correlation between cytokine expression and microbiota functions in RA

**Figure 7:**
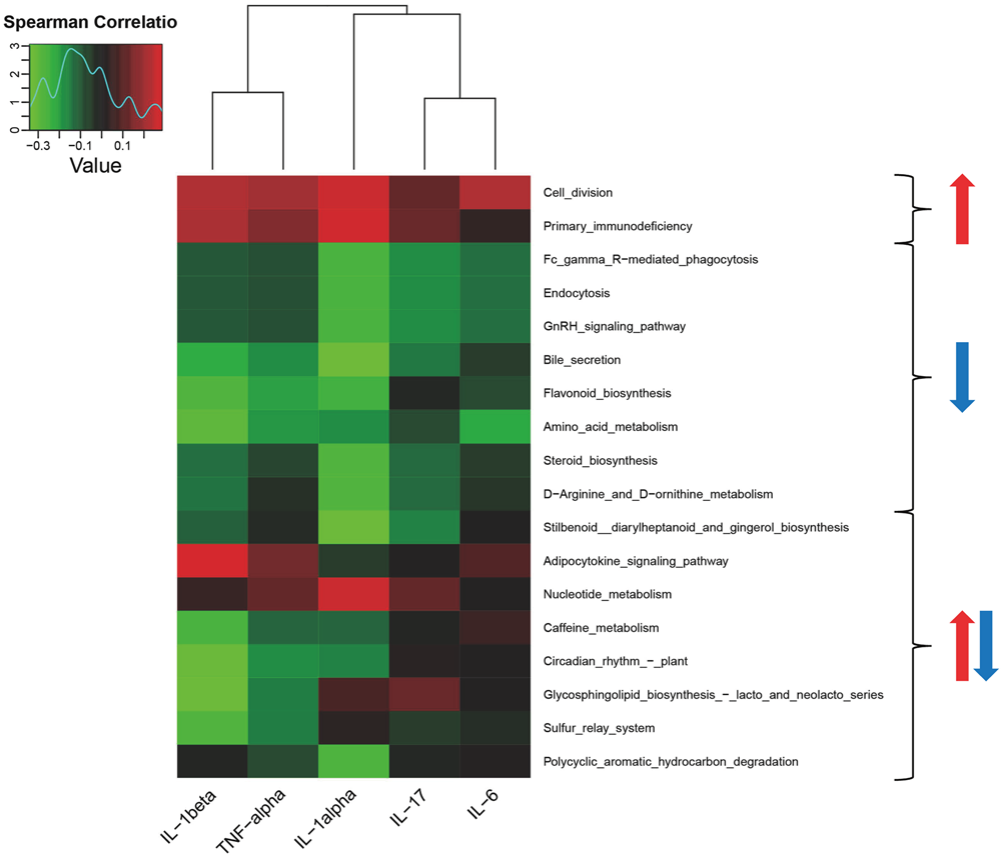
Heatmap showing correlations between cytokines and functions of microbiota in RA. Red arrows represent positive correlations, and blue arrows represent negative correlations.

We measured expression of five inflammatory factors – IL-1α, IL-1β, TNF-α, IL-6, and IL-17 – in blood samples of 58 RA patients and analyzed their correlations with microbiota functional analysis results from synovial fluid of these RA patients. Primary immune defects and cell proliferation were positively correlated with IL-1α, IL-1β, IL-6, and TNF-α expression. Fcγ receptor-mediated phagocytosis and endocytosis and the GnRH signaling pathway were negatively correlated with IL-17, IL-1α, and IL-6 expression. Bile secretion, flavonoid biosynthesis, and amino acid metabolism were negatively correlated with IL-1α, IL-1β, and TNF-α expression. The results are displayed in Fig. 7.

### RA and OA-associated microbiota in all synovial samples

**Figure 8:**
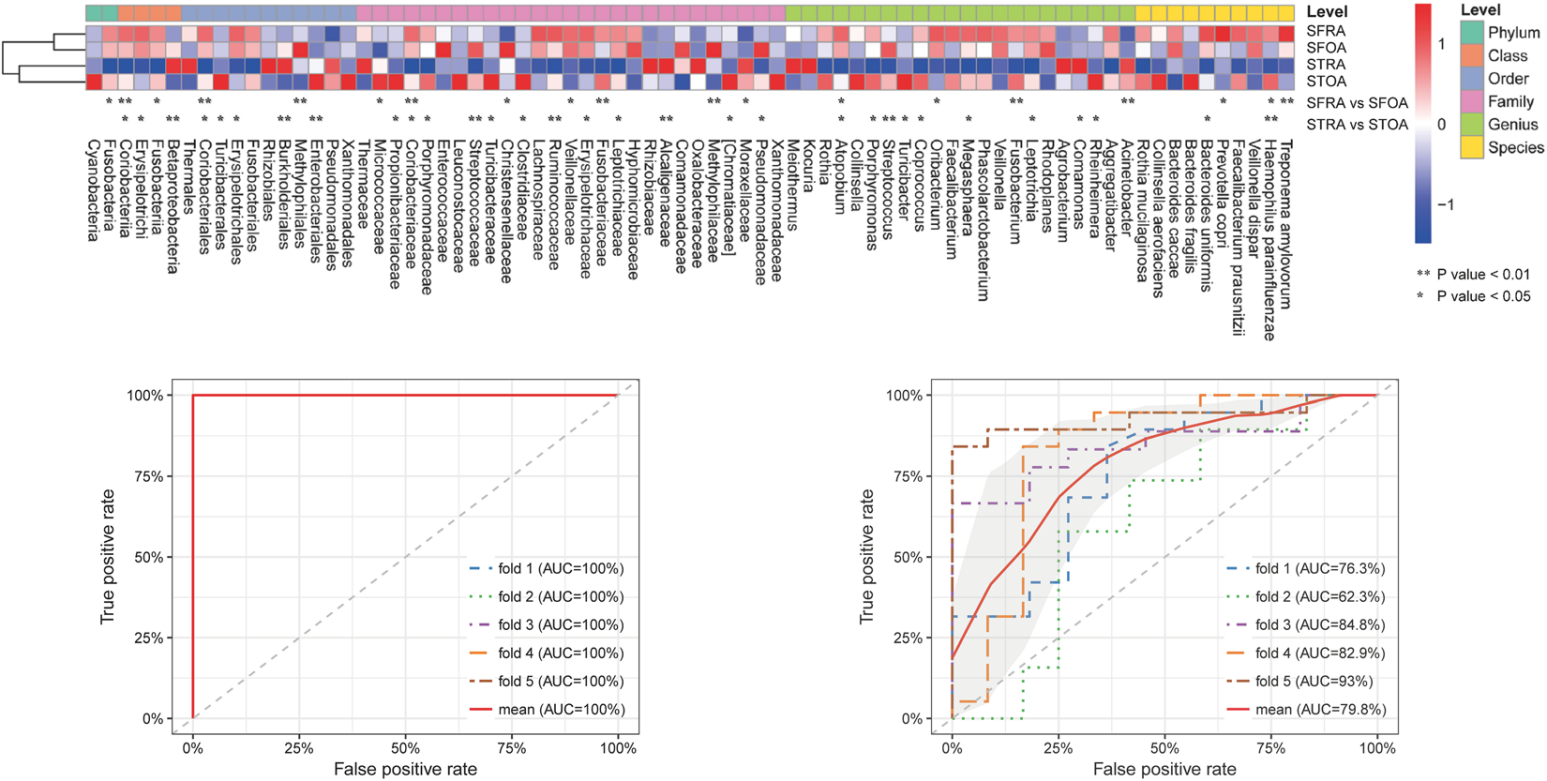
Microbiota associated with Rheumatoid arthritis and Osteoarthritis. (**A**) Relative abundance of the top 76 most different OTUs across taxonomic groups (*P* < 0.1, by Wilcoxon rank sum test). Abundance profiles were transformed into Z-scores by subtracting the average abundance and dividing the standard deviation of all samples. Z score was negative (shown in blue) when the row abundance was lower than the mean. *P < 0.01; **P < 0.05. (**B**) Receiver operating characteristic curves for STRA and STOA groups, determined using OTU markers, sequencing datasets, and a supervised learning approach with random Forest algorithm. (**C**) Receiver operating characteristic curves for SFRA and SFOA groups, determined using OTU markers, sequencing datasets, and a supervised learning approach with random Forest algorithm.

Relative abundance of microbiota was calculated by summing the abundance of data. *P*-values were tested by Wilcoxon rank sum test and corrected for multiple testing^16^. It is worth mentioning that 76 microbiotas were differentially enriched in RA and OA samples. The results are displayed in Fig. 8A. Genera such as *Fusobacterium* were overrepresented in SFRA samples. Using a machine learning approach, samples were mostly successfully classified into RA and OA groups, with the highest proportion of samples correctly classified when using OTU-level taxa along with data from RA and OA markers. With OTU-level markers of RA and OA, receiver operating curves predicted risk of arthritis. STRA samples were classified correctly and separately from STOA samples with a success rate of 1. The results are displayed in Fig. 8B. SFRA samples were classified correctly and separately from SFOA samples with a success rate of 0.86. The results are displayed in Fig. 8C.

## Discussion

Microbiomes are increasingly associated with the pathogenesis of RA, although most studies have focused on fecal and oral saliva samples^9–11^, whereas microenvironments in the articular cavity and lesions have not been systematically studied. On the other hand, OA appears to have a different etiology. Nonetheless, several phenomena in the OA acute phase response demonstrate the inflammatory properties of the disease and implicate various cytokines and chemokines in its pathogenesis^17–21^. However, whether infection is involved in OA pathogenesis remains controversial.

We assessed the bacterial nucleic acid in SF and ST of RA and OA patients with 16s rRNA gene amplicon sequencing. We found that the bacterial nucleic acid is different between RA and OA not only in SF but also in ST. Most importantly, we separately compared the profiles of bacterial nucleic acid between RA and OA in ST and SF, we then identified characteristic nucleic acid biomarkers in RA and OA.

Previous studies in SF and ST used traditional 16s rRNA gene PCR. Heijedn *et al*. used 16S-rRNA primers to detect the presence of bacterial DNA in RA (n=26), OA (n=5) and other form of arthritis samples^13^. DNA amplicons were also detected in the SF and/or ST samples from 5 patients with RA (5/26, 19.2%); these originated from multiple bacterial species. Martinez-Martinez *et al*., used Universal 16S-ribosomal RNA primers to detect the presence of bacterial DNA in SF and ST of patients with RA and other arthritis, and they used immunobiological analysis with a specific antibody to detect the presence of bacterial peptidoglycan–polysaccharide complexes in synovial tissue. Here, we aseptically collected ST and SF samples from patients with RA (n=125) and OA (n=58) and used 16S rRNA V1-V2 sequencing to analyze microbiome profiles. We found that both ST and SF possess microbial DNA amplicons belonging to various microflora, confirming that bacterial nucleic acids are present in the articular cavity of RA and OA. Compared with previous studies, we used a relatively larger cohort, and more importantly, our work is the first study on ST and SF in RA and OA patients using next-generation sequencing. The findings have been comprehensively validated, and significant variations in the diversity and relative abundance between RA and OA were further discovered. A limitation of this study is that we only confirm what bacterial nucleic acids exist in ST and SF, but we cannot prove there were bacteria in ST or SF. Future studies are required to directly confirm the same, using other methods.

A pathological feature of RA is synovial cell proliferation, which produces various cytokines. This inflammatory reaction results in synovial tissue hyperplasia, thus initiating a cycle that increases production of synovial fluid^22–25^. SF concentrates blood and ST components^26^, so we found significantly greater α-diversity in SFRA than STRA samples (*P* = 2.085e-06). In the pathology of OA, however, articular cartilage releases debris into the SF after destruction of pathogenic factors, stimulating synovitis. Synovitis releases inflammatory mediators, further degrading the cartilage and initiating a destructive cycle, while the amount of synovial fluid does not change significantly^27^. Therefore, STOA is not significantly affected by synovial erosion, and the α-analysis showed no significant difference between SFOA and STOA (*P* = 0.1689). These differences between RA and OA agree with existing literature, because although both lesion sites are ST, they are based on different pathological features^28,29^. Accordingly, we detected significantly different bacterial nucleic acids for STRA and STOA, with noteworthy aggregation (*P* = 0.00399).

A common pathological feature of RA and OA is synovial inflammatory response^30^. In early arthritis, it is difficult to clinically distinguish between RA and OA using imaging and histopathological methods^31,32^. Therefore DNA fragments of bacteria that assemble in the SF offer a distinguishing feature between STOA and STRA. Similar to intestinal bacteria, perhaps it is not complete change in flora that cause disease, but changes in the proportion of certain microbiota. Our results show no obvious aggregation in SFRA and SFOA samples (*P* = 0.03704). Further, our OTU data show that *Porphyromonas* and *Bacteroides* were present in all samples, and previous studies have found *Porphyromonas* in SF and serum of individuals with RA and in epithelial ulcer tissues of individuals with periodontitis^33,34^^.^

Taneja *et al*. showed that *HLA* expression affects intestinal flora and increases susceptibility of arthritis, suggesting that intestinal microbes can be used as biomarkers for RA research^33^. Our analysis of flora differences revealed that *Agrobacterium*, *Comamonas*, *Kocuria*, *Meiothermus*, and *Rhodoplanes* were concentrated in STRA samples, while *Atopobium*, *Phascolarctobacterium*, *Rhodotorula mucilaginosa*, *Bacteroides uniformis*, *Rothia*, *Megasphaera*, *Turicibacter*, *Leptotrichia*, *Haemophilus parainfluenzae*, *Bacteroides fragilis*, *Porphyromonas*, and *Streptococcus* were concentrated in STOA samples. Among them, *Haemophilus parainfluenzae*^9^ and *Porphyromonas*^35,36^ have been previously shown to be present in oral and gut microbiomes of individuals with arthritis.

Analysis of flora differences between SFRA and SFOA samples revealed that *Veillonella dispar*, *Haemophilus parainfluenzae*, *Prevotella copri*, *Fusobacterium*, and *Treponema amylovorum* are more abundant in SFRA samples, while *Bacteroides caccae* is concentrated in SFOA samples. Among them, *Haemophilus parainfluenzae*^9^, *Prevotella copri*^8,33,37^ and *Bacteroides caccae*^33,35–37^ have been previously shown to be present in feces of mouse models for arthritis. Our results show that these bacterial DNA are also present in ST or SF. Therefore, intestinal bacteria or oral bacteria may enter and settle into joint cavities through certain pathways and then become involved in the occurrence and development of arthritis.

Using function-module analysis with the KEGG database, we found consistent microbial flora function of ST and SF. Pathways related to metabolism (including amino acid metabolism, carbohydrate metabolism, energy metabolism, co-enzyme factor and vitamin metabolism, metabolism of terpenoids and ketones, and nucleotide metabolism) were positively correlated with microbiota function in the RA articular cavity, but negatively correlated with that in the OA articular cavity. Pathways and transcription factors related to genetic information processing were positively correlated with microbiota function in the OA articular cavity, but negatively correlated with that in the RA articular cavity. Therefore, some flora differences may play an important role in the pathophysiological mechanisms of RA or OA and may perhaps be directly involved in disease occurrence.

Clinical manifestations of RA include chronic synovitis and hyperplasia and fibrosis of synovial membranes^38^. In the interaction network of RA, inflammatory localized cytokines, IL-1 and TNF are located in the central area^39,40^. Subsequently, IL-6 and IL-7 are also involved in proliferation of synovial cells and induce production of matrix metalloproteinases, thereby aggravating destruction of articular cartilage^41,42^. In this study, we analyzed five inflammatory factors – IL-1α, IL-1β, TNF-α, IL-6, and IL-17 – in blood samples from 58 RA patients. At the same time, we functionally analyzed microbiota using 16S rRNA sequencing of the synovial fluid from these RA patients. Our results demonstrate primary immunologic deficiency and indicate that synovial cell proliferation – both pathological features of RA^22–25^ – are positively correlated with IL-1α, IL-1β, IL-6, and TNF-α expression. Our results also indicate that phagocytosis mediated by the Fcγ receptor – which plays a crucial role in RA pathogenesis^43–45^ – and the GnRH signaling pathway, are negatively correlated with IL-17, IL-1α, and IL-6 expression. Again, this indicates that the function of microbiota produced in synovial fluid affects inflammatory factors, although specific effects require further verification.

Further, *Fusobacterium* is one of the most common genera in human infections and can be found in body cavities of humans and other animals. *Fusobacterium nucleatum* has been reported in high numbers and frequency in patients with periodontitis^46^. Some reports have indicated that an infectious agent in a susceptible host could be one possible trigger factor for RA. It has been suggested that oral microorganisms and special periodontal bacteria (mainly *Porphyromonas gingivalis*), could be infectious agents^47^. Martinez-Martinez *et al*., found that 100% of patients showed periodontal bacterial DNA (PBDNA) in subgingival dental plaque (SDP) and synovial fluid (SF) and 83.5% in serum. *Prevotella intermedia* (89.4% and 73.6%) and *Porphyromonas gingivalis* (57.8% and 42.1%) were the species most frequently detected in SDP and SF, respectively^34^. Our results show that *Porphyromonas* exist in all SF samples (SFRA and SFOA) and *Fusobacterium* are overrepresented in SFRA samples. These results confirm that oral microorganisms may contribute to RA.

## Conclusions

The present research on microbial flora provides an excellent entry point to study various diseases. However, there is insufficient research on microbes related to the pathogenesis of RA and OA, impeding exploration of more effective and specific treatment methods. In this study, starting with synovial samples from arthritis lesions, we confirmed the presence of microbes in synovial tissue and synovial fluid and showed some microbial differences, which may be involved in the occurrence and development of RA and OA. With continuous development of molecular biology techniques, relationships of microorganisms with OA or RA can be better understood to provide a broader prospect for treatment of OA and RA. In addition, there are ongoing studies to further observe how microbes in synovial tissue and synovial fluid settle in joint cavities and accelerate the pathology of arthritis.

## Methods

### Ethics statement

This protocol was evaluated and approved by the Ethics Committee of Shandong Academy of Medicinal Sciences (Jinan City, Shandong). Our study was conducted according to the protocol guidelines of our institution and approved by the Ethics Committee of Shandong Academy of Medicinal Sciences (Jinan City, Shandong). All participants were adults (over 18 years old) and gave written informed consent and allowed their biological samples to be genetically analyzed.

### Study population

Our cohort was composed of 183 adult subjects; 125 were RA (STRA and SFRA) and 58 were OA (STOA and SFOA). The data is displayed in Table 1. Samples from the 183 subjects enrolled in this study were collected and analyzed at Qianfoshan Hospital, Jinan Central Hospital affiliated to Shandong University, and Guoyitang Hospital. A full list of sample information is available in Table S1. SF samples were collected aseptically during therapeutic aspiration from knee joints. ST were collected aseptically during joint surgery. Prevention of contamination and identification of controls – After excision, the fresh tissue was immediately placed in 50 ml sterile tubes on ice and homogenized within 10 min of collection, and the synovial fluid was placed in 5 ml sterile tubes on ice and homogenized within 5 min of collection. The negative controls included sample collection controls, reaction mixture controls and environmental control. For the environmental control, a tube filled with sterile phosphate-buffered saline (PBS) was left open for the duration of the surgical procedure and then processed in parallel with the samples. The whole experiment was strictly conducted in a sterile environment. Each sample was immediately frozen and stored at –135 °C without heparin and without hyaluronidase. Patients were excluded from the study if they had chronic infectious disease history, experienced an infection within 1 month before sampling, or received antibiotic therapy within 2 weeks before sampling.

**Table 1:** Demographic information of all subjects used in this study.

	Cohort	Synovial Fluid	Synovial Tissue
n	183	152	31
Cohort			
RA	125	110	15
OA	58	42	16
Male: Female	31:152	27:125	4:27
Age	20~80	20~80	22~78

Diagnosis of RA was performed by a rheumatologist in accordance with the criteria of the American College of Rheumatology, 2010^48,49^. Diagnosis of OA was performed based on the criteria of the American College of Rheumatology 2012 recommendations for the use of nonpharmacologic and pharmacologic therapies in osteoarthritis of the hand, hip, and knee^50^. No glucocorticoid therapy was performed within 3 months before admission; all patients were in the active period.

### DNA extraction and 16S rRNA sequencing

Bacterial genomic DNA extraction was performed with a DNeasy Blood & Tissue Kit (Qiagen) according to the manufacturer’s instructions. DNA quantitation was performed on a Nanodrop 2000 (Thermo Scientific). To generate 16S rRNA gene amplicons, 50 ng of DNA was used as a template in a 50-μL reaction, with 0.4 μM of V1-V2 barcoded primers targeting 27F and 355R of the bacterial 16S gene (5′-AGAGTTTGATCMTGGCTCAG-3′ and 5′-CTGCCTCCCGTAGGAGT-3′). Amplicons were purified with a QIAquick PCR Purification Kit (Qiagen). All amplicons were quantified and then pooled to equalize concentrations for sequencing on an Illumina HiSeq.

### 16S rRNA sequence analysis

The 16S sequence paired-end data set was joined and quality filtered using the FLASH method described by Magoč and Salzberg^51^. All sequence analysis was performed in the Quantitative Insights into Microbial Ecology (QIIME, version 1.9.1) software suite^52^ according to the Qiime tutorial (http://qiime.org), with some modified methods. Chimeric sequences were removed using usearch61 with denovo models^53^. Sequences were clustered against the 2013 Greengenes (13_8 release) ribosomal database’s 97% reference data set. Sequences that did not match any entries in this reference were subsequently clustered into de novo OTUs at 97% similarity with UCLUST. Taxonomy was assigned to all OTUs using the RDP classifier^54^ within QIIME and the Greengenes reference data set. Rarefaction and rank abundance curves were calculated from OTU tables using alpha diversity and rank abundance scripts within the QIIME pipeline. Hierarchical clustering based on population profiles of most common and abundant taxa was performed using UPGMA clustering (unweighted pair group method with arithmetic mean, also known as average linkage) on the distance matrix of OTU abundance. The resulting newick formatted tree was obtained with the QIIME package.

### Cytokine expression

We used an ELISA kit (Anhui Joyee Biotechnics Co. Ltd.) to quantitatively analyze expression of human IL-1α, IL-1β, TNF-α, IL-6, and IL-17. An ELISA assay was then performed according to the manufacturer’s instructions.

### Statistical analysis

To account for any bias caused by uneven sequencing depth, the least number of sequences present in any given sample from a sample category were randomly selected prior to calculating community-wide dissimilarity measures (α-diversity and β-diversity). Diversity analysis was performed using rarefied OTU tables (rarefied to the lowest number of reads obtained for any of the samples analyzed). All PCoA were based on unweighted and weighted UniFrac distances using evenly sampled OTU abundances. Prediction of the functional composition of a metagenome using marker gene data and a database of reference genomes was done with PICRUSt as described by Langille *et al*.^55^. Graphical representation of the results was done with R. Wilcox rank sum test was used to calculate *P*-values. Classification analysis was performed by Support Vector Machine (SVM) with R package e1071. For each classification task, the radial basis function (RBF) was chosen as the kernel function, and the best values of the two parameters, cost (C) and gamma (γ) in the kernel function were obtained by a grid-search approach using cross-validation. Finally, the classification accuracy was evaluated by five-fold cross-validation. The ROC curves as well as the AUC (Area under the Curve) value was calculated using the ROCR R package.

## Electronic supplementary material

Dataset 1

Supplementary information

## Data Availability

The datasets generated during and/or analyzed during the current study are available from the corresponding author on request.
