# Supplementary material for: Detection and characterization of bacterial nucleic acids in culture-negative synovial tissue and fluid samples from rheumatoid arthritis or osteoarthritis patients
Source: Sci Rep. 2018 Sep 24;8:14305. doi: 10.1038/s41598-018-32675-w (PMC6155189; doi:10.1038/s41598-018-32675-w)
Supplement: Supplementary file 2 — Supplementary information [file 41598_2018_32675_MOESM2_ESM.pdf]

Supplementary Information for “Detection and characterization of bacterial nucleic acids in culture-negative synovial tissue and fluid samples from rheumatoid arthritis or osteoarthritis patients”

Yan Zhao[1, 2, #], Bin Chen[1, #], Shufeng Li[3], Lanxiu Yang[4], Dequan Zhu[5], Ye Wang[6], Haiying Wang[1], Tao Wang[2], Bin Shi[2], Zhongtao Gai[7], Jun Yang[10], Xueyuan Heng[5*], Junjie Yang[1, 8, *], Lei Zhang[1, 5, 6, 7, 9, 10, *]

[1]College of Life Science, Shandong Normal University, Jinan 250014, China. [2]Shandong Medicinal and Biotechnology Centre, Shandong Academy of Medical Sciences, Jinan 250062, China. [3]Department of Orthopedics, Qianfoshan Hospital Affiliated to Shandong University, Jinan 250014, China. [4]Guoyitang Hospital, Jinan 250000, China. [5]Microbiological Laboratory, Linyi People’s Hospital, Linyi 276003, China. [6]Qingdao Human Microbiome Center, The Affiliated Central Hospital of Qingdao University, Qingdao 266000, China. [7]Shandong Children’s Microbiome Center, Qilu Children's Hospital of Shandong University, Jinan 250001, China. [8]College of Life Science, Qilu Normal University, Jinan 250200, China. [9]Shandong Institutes for Food and Drug Control, Jinan 250101, China. [10]Beijing Advanced Innovation Center for Big Data-Based Precision Medicine, School of Chemistry and Environment, Beihang University, Beijing 100191, China.

# Yan Zhao and Bin Chen contributed equally to this work.

* Correspondence and requests for materials should be addressed to Lei Zhang (email: microbiome@foxmail.com), or Junjie Yang (email: microbiota@foxmail.com), or Xueyuan Heng (email: xueyuanheng@yahoo.com)

Microbial cultivation

All 2g ST and 5ul SF samples were inoculated in different culture media, (LB medium, CTFUD medium with glucose, cellobiose and sucrose as carbon resource, respectively, Wheat Bran Medium, LB medium with pH7.6). They were incubated under aerobic and anaerobic conditions at 37℃ for 24, 48, 72, 96 and 108 h. Reference strains were used as positive controls in each experiment set. The isolated bacteria were verified by sequencing comparison.

We found that *clostridium* was positive in the synovial tissue of 2 patients with STOA and 1 patient with STRA. The complete 16S rRNA gene of these cultured strains were sequenced and could be matched to the high-throughput 16S rRNA sequencing results (*Clostridium botulinum* strain CDC_1632 and *Clostridium sporogenes* strain CUA1 in STOA, *Clostridium botulinum* strain CDC_1632 and *Clostridium Clostridium* sp. K14 in STRA). The results are displayed in Figure S1.

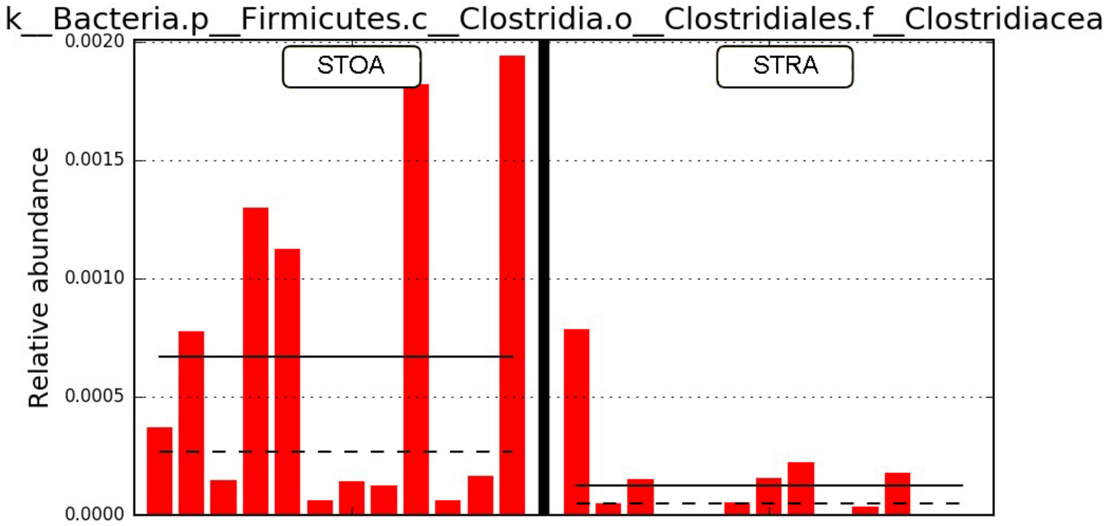

Figure S1: *clostridiacea* exist in all STOA samples (100%) and exist in 8 STOA samples (61.5%)

Electron microscopy and laser scanning confocal microscopy analysis of anaerobic bacteria in SF samples.

The SF sample is filtered through the membrane (special membrane for flow cytometry) to remove solids and large particles (Filtrate A).

Filtrate A was filtered through a 0.45 um membrane to remove most human cells (Filtrate B).

Filtrate B was filtered through a 0.22 um filters membrane to enrich bacteria and then washed with sterilized ultra-pure water to obtain Filtrate C.

Filtrate C was observed using scanning electron microscope and laser scanning confocal microscopy. The results are displayed in Figure S2.

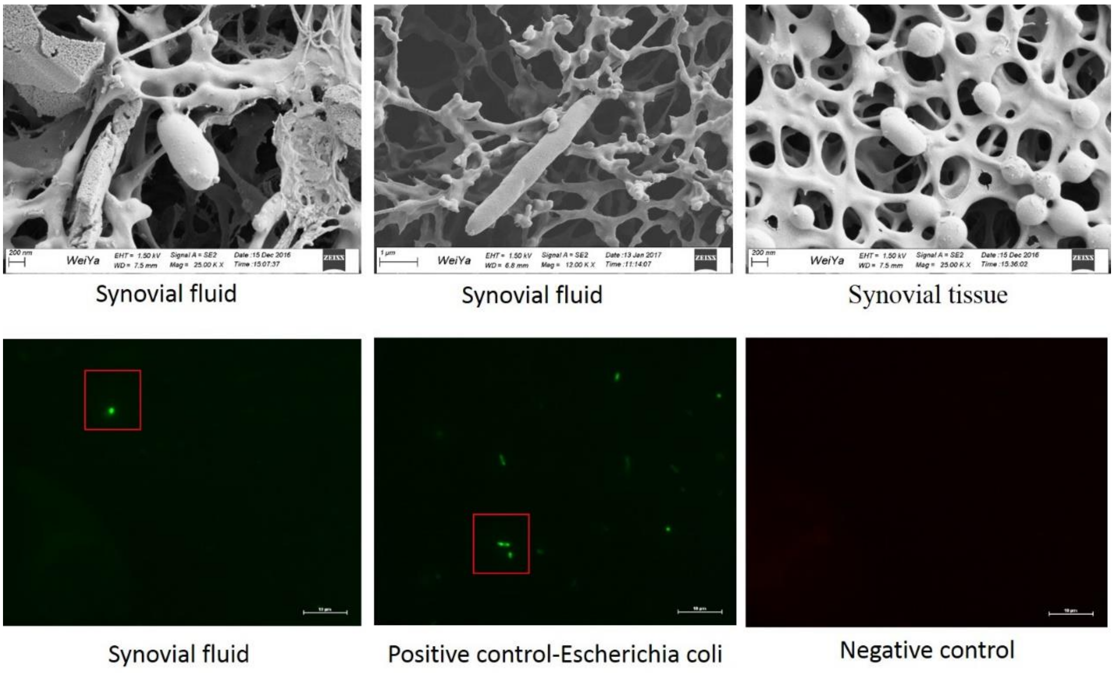

Figure S2: Observed suspected bacteria based on electron microscopy were 0.75 microns and observed suspected bacteria based on laser scanning confocal microscopy were 0.75 microns. (SYTO™ 9 Green Fluorescent Nucleic Acid Stain, 630 x)

Discrimination between rheumatoid arthritis and osteoarthritis.

The pathogenesis of the two diseases is basically different. The pathogenesis of osteoarthritis usually belongs to primary or secondary disease, and cartilage degeneration of the patient causes the disease. Rheumatoid arthritis is based on the patient's systemic autoimmune disease.

There are also many different pathogenesis for the two diseases. The main lesions in osteoarthritis are located in the cartilage of the patient and degeneration of the bone and joint leads to secondary synovitis. Bone hyperplasia in the patient leads to

osteophyte joints. When the osteophyte is heavier, the cartilage and the lower bone will be seriously damaged. The main component of the disease of rheumatoid arthritis is in the synovial membrane of the patient. When the synovial membrane of the patient is formed, the pannus will cause the patient's cartilage to suffer certain erosive damage.

The age of onset for patients of the two diseases is different. Osteoarthritis is common in elderly patients over the age of 50, and the incidence rate is as high as 80%. The occurrence of this disease is related to the patient's age, sex and body weight. In patients younger than 50 years of age, the incidence in male patients is significantly higher than that in female patients; in case of incidence after the 50 years of age, the age of males is greater than that of females. The age of onset of rheumatoid arthritis is 30-50 years old. It is generally found in younger patients.

There are also many differences in the patient's joint involvement. Osteoarthritis often causes some erosion in the joints and knee joints of the patients, but rarely attacks the distal joints. However, rheumatoid arthritis is more common in palm joints, wrist joints and proximal knuckles of patients, and appears to have a more serious affect.

The disease has many different effects on the patient's body. Osteoarthritis is often only present in the patient's bones and joints, but does not cause secondary diseases in other parts of the body, blood vessels, etc.; Rheumatoid arthritis is often manifested in many small joint organs.

The results of X-ray examination of the two diseases are different. Osteoarthritis is often more serious with osteophyte, and the gap between the joints of the patient is relatively narrow, however, rheumatoid arthritis often leads to erosion, and the joint of the patient is deformed.

There are many differences in the synovial mechanism between the two diseases, and there are many differences in the genetics of the two diseases. There is no significant difference in the location of each disease locus in osteoarthritis, while rheumatoid arthritis is associated with HLA-DR4.
